# Substrates of Opposite Polarities and Downstream Processing for Efficient Production of the Biosurfactant Mannosylerythritol Lipids from *Moesziomyces* spp.

**DOI:** 10.1007/s12010-023-04317-z

**Published:** 2023-02-22

**Authors:** Nuno Torres Faria, Miguel Figueiredo Nascimento, Flávio Alves Ferreira, Teresa Esteves, Marisa Viegas Santos, Frederico Castelo Ferreira

**Affiliations:** 1 Department of Bioengineering and iBB - Institute for Bioengineering and Biosciences, Instituto Superior Técnico, Universidade de Lisboa, Av. Rovisco Pais, 1049-001 Lisbon, Portugal; 2 Associate Laboratory i4HB – Institute for Health and Bioeconomy, Instituto Superior Técnico, Universidade de Lisboa, 1049-001 Lisbon, Portugal

**Keywords:** Biosurfactant, Mannosylerythritol lipids, Co-substrates, Downstream processing, Nanofiltration

## Abstract

Biosurfactants can replace fossil-driven surfactants with positive environmental impacts, owing to their low eco-toxicity and high biodegradability. However, their large-scale production and application are restricted by high production costs. Such costs can be reduced using renewable raw materials and facilitated downstream processing. Here, a novel strategy for mannosylerythritol lipid (MEL) production explores the combination of hydrophilic and hydrophobic carbon sources sideways with a novel downstream processing strategy, based on nanofiltration technology. Co-substrate MEL production by *Moesziomyces antarcticus* was threefold higher than using D-glucose with low levels of residual lipids. The use of waste frying oil instead of soybean oil (SBO) in co-substrate strategy resulted in similar MEL production. *Moesziomyces antarcticus* cultivations, using 3.9 M of total carbon in substrates, yields 7.3, 18.1, and 20.1 g/L of MEL, and 2.1, 10.0, and 5.1 g/L of residual lipids, for D-glucose, SBO, and a combination of D-Glucose and SBO, respectively. Such approach makes it possible to reduce the amount of oil used, offset by the equivalent molar increase in D-glucose, improving sustainability and decreasing residual unconsumed oil substrates, facilitating downstream processing. *Moesziomyces* spp. also produces lipases that broken down the oil and, thus, residual unconsumed oils are in the form of free fatty-acids or monoacylglycerol, which are smaller molecules than MEL. Therefore, nanofiltration of ethyl acetate extracts from co-substrate-based culture broths allows to improve MEL purity (ratio of MEL per total MEL and residual lipids) from 66 to 93% using 3-diavolumes.

**Supplementary Information:**

The online version contains supplementary material available at 10.1007/s12010-023-04317-z.

## Introduction

The development of cleaner and sustainable production processes, along with lower residues generation, is highly necessary. Several companies have emerged and grown since the industrial revolution, especially the chemical industry, through the extraction of petroleum and the production of several compounds used in various applications. Surfactants are an example of molecules derived from petroleum. They are surface-active agents composed by a hydrophobic tail and a hydrophilic head, known as amphipathic structure. These compounds have unique properties and are used in a wide range of products, such as detergents, household products, and motor oils. The surfactant market is expected to reach 39.9 USD billion in 2021 [1].

Biosurfactants have the potential to replace fossil-driven surfactants with positive environmental impacts owing to their low eco-toxicity, high biodegradability rate, tolerance to high temperatures, pH and salinity, and mild production conditions [2]. Examples of biosurfactants include the glycolipids: sophorolipids, rhamnolipids, and mannosylerythritol lipids (MELs). This class of molecules started to be commercialized as components of sustainable cleaning product solutions [3]. However, the large-scale production of biosurfactants has been challenged by the high production costs. In this regard, the use of renewable raw materials, high product titres, and facilitated downstream processing are highly desirable to reduce the overall production costs [2].

**Fig. 1:**
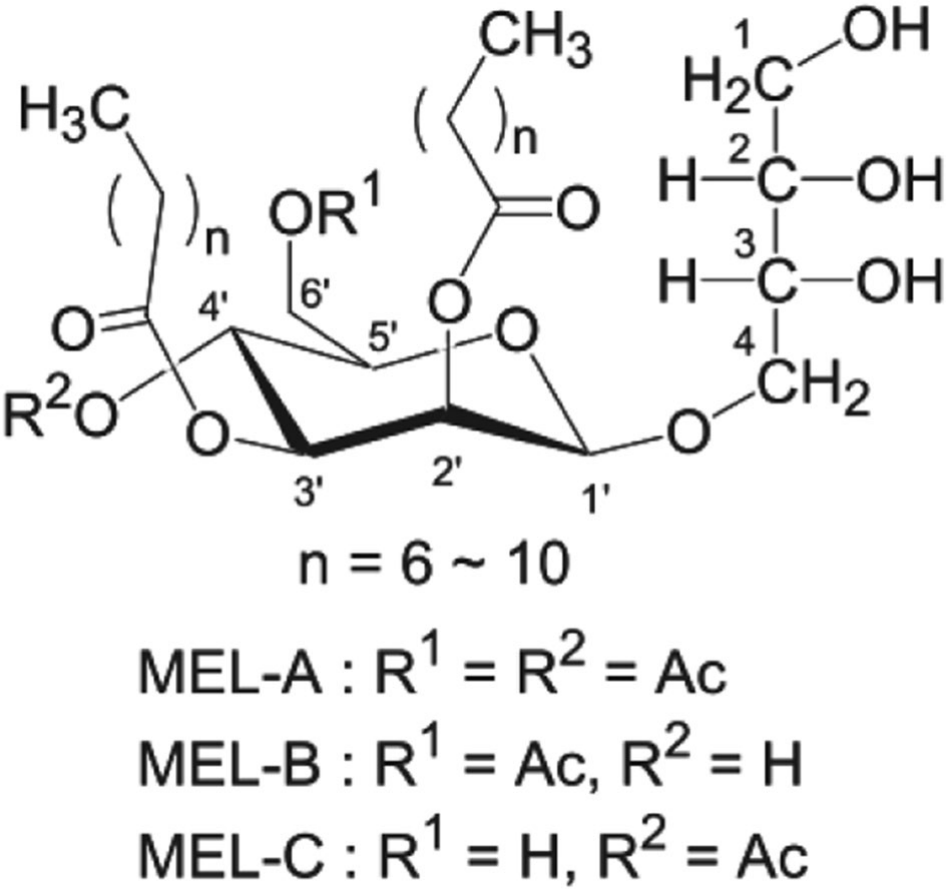
Chemical structure of MEL and their types. MEL-A: di-acylated; MEL-B: mono-acylated in C6; MEL-C: mono-acylated in C4

MELs are produced by microorganisms such as *Ustilago maydis* [4] and *Moesziomyces* (former *Pseudozyma*) *genus*, especially *M. antarticus*, *M. rugulosus*, and *M. aphidis*. MELs contain 4-O-β-D-mannopyranosyl-meso-erythritol as the hydrophilic group and fatty acid short-chains, as the hydrophobic group (Fig. 1) [5]. According to the number and position of the acetyl group, MELs are classified as MEL-A, di-acylated congener; MEL-B, mono-acylated congener in C6; MEL-C, mono-acylated congener in C4 and MEL-D, deacylated congener [6]. Other factors influence the structure of MEL, such as the number of acylation in mannose, the fatty acid length, and their saturation. These surface active molecules have shown relevant properties, such as the induction of differentiation of human promyelocytic leukaemia cell line HL60 [7] or the ability to downregulate tyrosine kinase in K562 cells, inhibiting the cell proliferation and inducing differentiation [8]. Also, recent studies demonstrated the potential effect of MEL to treat skin injuries [9], showing moisturizing activity [10, 11], as well as hair repair properties [12]. Furthermore, it was also tested as biopesticide [13, 14] and food preservative, with application of MELs in breadmaking industry, due to the avoidance of microbial spoilage [15, 16].

Different carbon sources have been tested to increase MEL production. In general, carbon sources for MEL production can be divided into two main groups: hydrophilic and hydrophobic carbon sources. Vegetable oils, and more specifically soybean oil (SBO), have been a reference hydrophobic carbon source used to produce MEL, either with *M. antarcticus* T-34 [17], or *M. aphidis* DSM 14,930 [18]. The yield of 0.92 g_MEL_/g_substrate_ is the highest reported, but no reference to the total SBO used and the final composition of the broth, regarding the presence of residual lipids, was provided [19]. Recently, Beck et al. [20] have developed a bioreactor fed-batch process using *M. aphidis* in defined mineral salt media, obtaining 50.5 g/L of MELs when vegetable oil was added in excess with consequent high impurities, while a concentration of 34.3 g/L and 0.294 g_MEL_/g_substrate_ were obtained, after 170 h, by adjusting the oil feed, improving the extract purity (around 90%).

The separation of residual oil requires complex processes using multi-steps extraction by different organic solvents [21], turning their industrial implementation cumbersome and yielding complex solvents mixtures of difficult recyclability.

The problems associated with downstream processing of MEL when vegetable oils are used can be circumvented using hydrophilic carbon sources. Morita et al. [22], demonstrated that *M. antarcticus* T-34 is able to produce MEL from D-glucose. MEL production from sugar pentoses (xylose and arabinose) was also demonstrated using *M. antarcticus* PYCC 5048^ T^, *M. aphidis* PYCC 5535^ T^, and *M. rugulosa* PYCC 5537^ T^ [5]. Pre-treated lignocellulosic residues by an enzymatic process were fed to *M. antarcticus* PYCC 5048^ T^ and *M. aphidis* PYCC 5535^ T^ to produce MEL [23]. Other hydrophilic sources have been recently assessed, such as the use of coconut water [24], cassava wastewater [25], or cheese whey [26]. While these studies using hydrophilic carbon sources report low accumulation of residual lipids, the titres obtained are still low.

Therefore, the current study explores MEL production based on a combination between hydrophilic and hydrophobic carbon sources. This approach aims at improve MEL titres obtained using lignocellulose material (LCM)-based sugars [5, 27], while improving downstream processing [18]. The hydrophilic carbon source is used to produce biomass and lipases, assuring that any residual unconsumed oil substrates will be present as free fatty acids, of smaller molecular weight than MEL, and complemented with hydrophobic carbon source, used to trigger MEL production. Complementary, a downstream strategy for MEL purification, based on molecular weight of MEL and residual unconsumed lipids, was assessed, through the development of a diafiltration process with nanofiltration membranes.

## Materials and Methods

### Microorganisms and Maintenance

*Moesziomyces* yeast strains were provided by the Portuguese Yeast Culture Collection (PYCC), UCIBIO/Requimte, FCT/UNL, Caparica, Portugal: *M. antarcticus* PYCC 5048 T (CBS 5955) and *M. aphidis* PYCC 5535 T (CBS 6821). These strains were plated in YM agar (yeast extract 3 g/L, malt extract 3 g/L, peptone 5 g/L, D-glucose, and agar 20 g/L) and incubated for 3 days at 27 °C. Stock cultures were prepared by propagation of yeast cells in liquid medium, as described below for the inoculum, and stored in 20% v/v glycerol aliquots at − 80 °C for further use. These stocks are renewed every 6 months.

### Media and Cultivation Conditions

The production of MEL was initiated with the preparation of an inoculum by transferring the yeast colonies of *M. antarcticus* and *M. aphidis* into an Erlenmeyer flask with one-fifth working volume of medium (50 mL), as reported by Faria et al. [5]. These flasks contained 3 g/L NaNO_3_, 0.3 g/L MgSO_4_, 0.3 g/L KH_2_PO_4_, 1 g/L yeast extract, and 40 g/L D-glucose, and incubated in the orbital at 27 °C with 250 rpm, for 48 h. The cell cultivation was initiated with 10% (v/v) of inoculum added into an Erlenmeyer flask with one-fifth working volume (50 mL) containing 0.3 g/L MgSO_4_, 0.3 g/L KH_2_PO_4_, 1 g/L yeast extract, 3 g/L NaNO_3_ and carbon source, either hydrophilic (D-glucose), or hydrophobic (SBO or waste frying oil) with different concentrations and proportions (described in the sections below), for 14 days at 250 rpm and 27 °C.

### Cultivation Conditions on Co-substrate Strategy Development

Three strategies were here tested in *M. antarcticus* and *M. aphidis* cultivations: (i) the use of SBO (OliSoja, Portugal) (from 20 to 80 g/L) sole carbon source; (ii) the use of D-glucose as sole carbon source, starting the culture with D-glucose (40 g/L) and further addition at day 4 of cultivation of D-glucose (40 or 80 g/L); and (iii) co-substrate strategy, starting the culture with D-glucose (40 g/L) and with further supplementation at day 4 of cultivation of SBO waste fried oil, in different proportions, to a total carbon added in cultivation of 2.6 and 3.9 M.

### Growth and Biomass Determination

Cellular growth was followed by measuring cell dried weight (CDW). CDW was determined from 1 mL of culture broth by centrifugation at 13,000 rpm for 5 min, followed by cell pellet washing with 500 µL of deionized water (twice) and drying at 60 °C for 48 h.

### High-Performance Liquid Chromatography Analysis

Culture broth samples were centrifuged at 13,000 rpm for 5 min, and the supernatants filtered through a 0.22-µm-pore size-filter. D-glucose quantification was performed using a system Merck Hitachi, Darmstadt, Germany) equipped with a refractive index detector (L-7490, Merck Hitachi, Darmstadt, Germany) and an Rezex ROA Organic Acid H^+^ column (300 mm × 7.8 mm, Phenomenex, Torrance, CA, USA), at 65 °C. Sulfuric acid (5 mM) was used as mobile phase at 0.5 mL/min. The concentrations of monoglycerides (MAG), diglycerides (DAG), and triglycerides (TAG) in the initial solution (feed), permeate, and retentate were analysed by HPLC, as described by elsewhere [28]. The HPLC was equipped with a Chromolith Performance RP-18 endcapped (100 mm × 4.6 mm × 2 μm) column, an auto sampler (Hitachi LaChrom Elite L-2200), a pump (Hitachi LaChrom Elite L-2130), and a UV detector (Hitachi LaChrom Elite L-2400) set up at 205 nm. The flow rate was set up at 1 mL/min, and the injection volume was 20 μL. Three mobile phases were employed: phase A consisted of 100% acetonitrile, phase B consisted of water 100%, and phase C comprising a mixture of n-hexane and 2-propanol (4:5, v/v). Quantification was carried out using calibration curves of glyceryl trioleate (~ 65%, Sigma-Aldrich GmbH) for TAG, 1,3-Diolein (≥ 99%, Sigma-Aldrich GmbH) and 1-oleoyl-rac-glycerol (≥ 99%, Sigma-Aldrich GmbH) for MAG.

### Gas Chromatography Analysis

During the fermentations, 1 mL of culture broth was periodically taken and freeze-dried. The fatty acid content of the biological samples was determined by methanolysis and GC analysis of methyl esters as described by Welz et al. [29]. Initially, pure methanol (20 mL) was cooled down to 0 °C under nitrogen atmosphere and acetyl chloride (1 mL) was added under stirring over 10 min to generate a water-free HCl/methanol solution. Culture broth samples, after freeze-drying, were weighted and mixed with 2 mL HCl/methanol solution (0.67 N of HCl) and 100 µL of internal standard, 4% (v/v) heptanoic acid and 96% (v/v) of n-Hexane. Then, the samples were incubated for 1 h at 80 °C for reaction into methyl esters. The resulting product was extracted with 1 mL of n-hexane and 1 mL of water. The organic phase was retrieved, and 1 µL was injected in a GC system (Hewlett-Packard, HP5890), equipped with a FID detector and an Agilent HP-Ultra2 capillary column (L 50 m × I.D. 0.32 mm, df 0.52 µm). The oven was programmed from 140 °C, and temperature was raised to 170 °C at 15 °C/min, to 210 °C at 40 °C/min, and to 310 °C at 50 °C/min with a final isothermal plateau at 310 °C for 3 min. Carrier gas (nitrogen) was used with a split ratio of 1/25. MEL was quantified through the amount of C8, C10, and C12 fatty acids as previously described [5].

### Membrane Preparation

A home-made polybenzimidazole (PBI) membrane was manufactured by traditional phase inversion technique following previous protocol [30]. Celazole ® S26 solution (26 wt% PBI, 1.5 wt% LiCl in DMAc, PBI Performance Products Inc., USA) was diluted with N,N-dimethylacetamide (DMAc) (Panreac, Spain) to 22 wt% PBI concentration. The solution was mechanically stirred at 60 RPM overnight to obtain a homogeneous dope solution, which was then left still for 24 h for air bubbles removal. The resulting solution was first manually casted using a home-made casting knife height of 250 µm on the top of a non-woven Polyolefin Novatexx 2471 (Freudenberg Filtration Technologies, Germany), then immersed in a distilled water precipitation bath (1 h, three times) and then in an isopropanol (Carlo Erba, Spain) bath (1 h, three times) for water removal and kept on isopropanol until to be used. All the processes were performed at room temperature. The membrane was not crosslinked and used directly for nanofiltrations.

### Nanofiltration of Mannosylerythritol Lipid Extracts

A dead-end Sterlitech HP 4750 Stirred Cell fitted with a circular piece of the home-made PBI membrane with an area of 14.6 cm^2^ was used to carry out the filtrations. Replicate were performed using different membrane pieces. A pressure was applied using nitrogen, providing the driving force for the filtrations. All experiments were performed under magnetic stirring of 300 rpm and assays only performed after membrane preconditioned by filtering pure solvent, until a constant solvent flux was obtained, at room temperature.

Solutions of polystyrene oligomer 580 Da (Agilent Technologies, UK) and Rose Bengal 1017 Da (Sigma-Aldrich, Switzerland) were prepared for membrane characterization. Typical procedure to recover MEL from the cell culture broth consists of ethyl acetate (EtOAc) extraction of the whole culture broth (1:1 vol) twice, separation of phase, and evaporation of the organic phase to yield crude MEL — an orange gum enriched in MEL. Therefore, nanofiltrations were fed with a solution of crude MEL dissolved on 50 mL of EtOAc.

Nanofiltrations on concentration mode to 50% the initial volume was preformed to estimate rejection values (R) using Eq. 1 based on solute concentration in feed (*Cf*) and permeate (*Cp*). Such assays were performed first for polystyrene oligomer and Rose Bengal on acetonitrile (Fisher Chemicals, USA) and then for MEL and residual lipids in EtOAc.

1 $$R=\left(1-\frac{Cp}{Cf}\right)\times 100$$

A diafiltration strategy was then performed to purify MEL, retaining this molecule while pushing the lipidic molecules through an organic solvent nanofiltration (OSN) membrane. Again, the diafiltration was started by adding 50 mL of contaminated MEL in EtOAc solution and, using an HPLC pump Series I, Scientific Systems Inc., fresh EtOAc was add as required to keep cell volume constant, compensating for the volume leaving the system through the permeate. Samples were collected after addition of 3 and 6 diavolumes, with one diavolume corresponding to 50 mL, of EtOAc.

## Results and Discussion

*Moesziomyces* spp. have been described as MEL producers. They can use different carbon sources, ranging from vegetable oils to hydrophilic sugars, at high substrate concentration, either in batch or fed-batch culture mode [6]. The carbon source influences MEL titres, yields, and the purification steps needed for product recovery. The work here presented aims at two important aspects of MEL production intrinsically related: the combination of selected substrate able to increase biosurfactant yield and the decrease of residual lipids/MEL ratio, facilitating downstream processing.

### Mannosylerythritol Lipid Production and Residual Lipids Obtained from Soybean Oil or D-glucose

The increase of substrate concentration is a common strategy to improve biosurfactant titres. However, a high load of vegetable oils often results in high amounts of residual and/or unconsumed lipids, hampering biosurfactant purification [19].

**Fig. 2:**
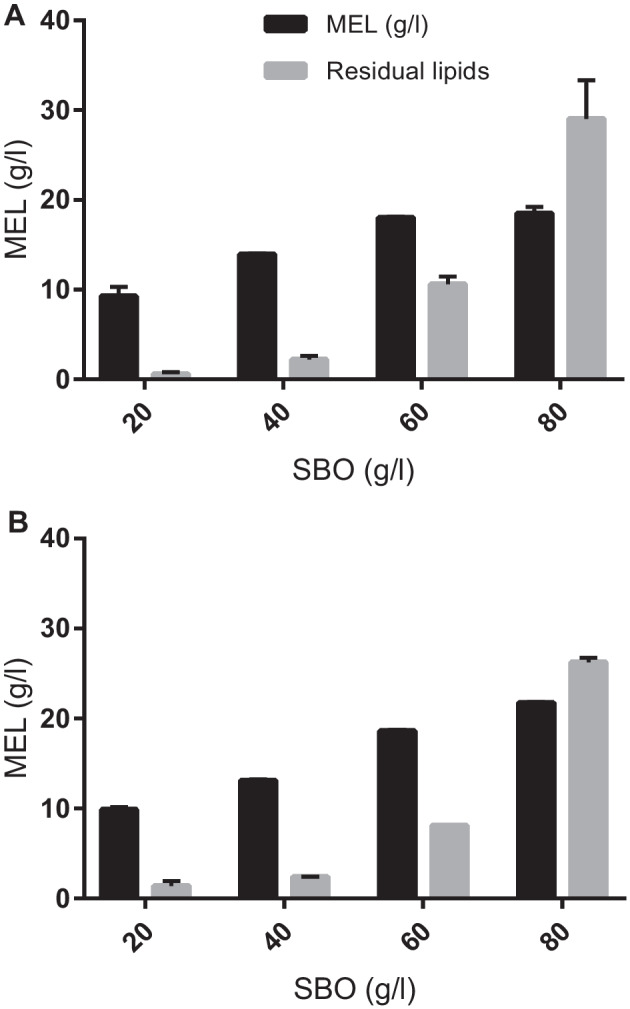
MEL and residual lipids obtained after 14 days cultivation of *M. antarcticus* and *M. aphidis* on SBO as sole carbon

*Moesziomyces* spp. cultivations using SBO were performed to stablish a baseline for the ability of the yeasts used to produce MEL. Yields and purity, as well as substrate utilization and product/residues formation, were assessed (Table 1; Fig. 2). Although purity definition often considers all form of contaminants, here purity is presented as a ratio of MEL to the sum of MEL and total residual lipids, major contaminants after MEL liquid–liquid extraction. The MEL titres obtained after 14 days cultivation of *M. antarcticus* and *M. aphidis*, using 20 to 80 g/L of SBO, increased non-linearly: *M. antarcticus* produced 9 to 20 g/L, respectively; *M. aphidis* produced 8 to 20 g/L, respectively. The residual lipids increased from 1 to 29 g/L, respectively, in *M. antarcticus* cultivations, and 1 to 27 g/L, respectively, in *M. aphidis* cultivations. Consequently, the higher the amount of SBO used, the lower was the purity: in *M. antarcticus* cultivation decreased from 92% (w/w) to 40% using 20 and 80 g/L, respectively, of SBO.

**Table 1:** *Moesziomyces antarcticus* and *M. aphidis* cultivation using soybean oil as sole carbon source (20, 40, 60, and 80 g/L): yields, titres, maximum dry biomass, and productivity

	Condition (SBO g/L)	Carbon in substrate (M)	MEL (g/L)	Yield of MEL/substrate (g/g)	Residual lipids (g/L)	Yield of residual lipids/substrate (g/g)	Purity (g/g)
*M. antarcticus*	80	5.2	19.5	0.24	29.1	0.40	0.37
	60	3.9	18.1	0.30	10.0	0.13	0.64
	40	2.6	14.0	0.35	1.9	0.02	0.88
	20	1.3	9.3	0.50	0.8	0.01	0.92
*M. aphidis*	80	5.2	21.8	0.27	26.6	0.33	0.45
	60	3.9	18.7	0.231	8.1	0.10	0.70
	40	2.6	13.2	0.33	2.4	0.03	0.85
	20	1.3	9.9	0.50	1.4	0.02	0.88

Purity (g/g) — ratio of g of MEL to the sum of g of MEL and residual lipids

**Fig. 3:**
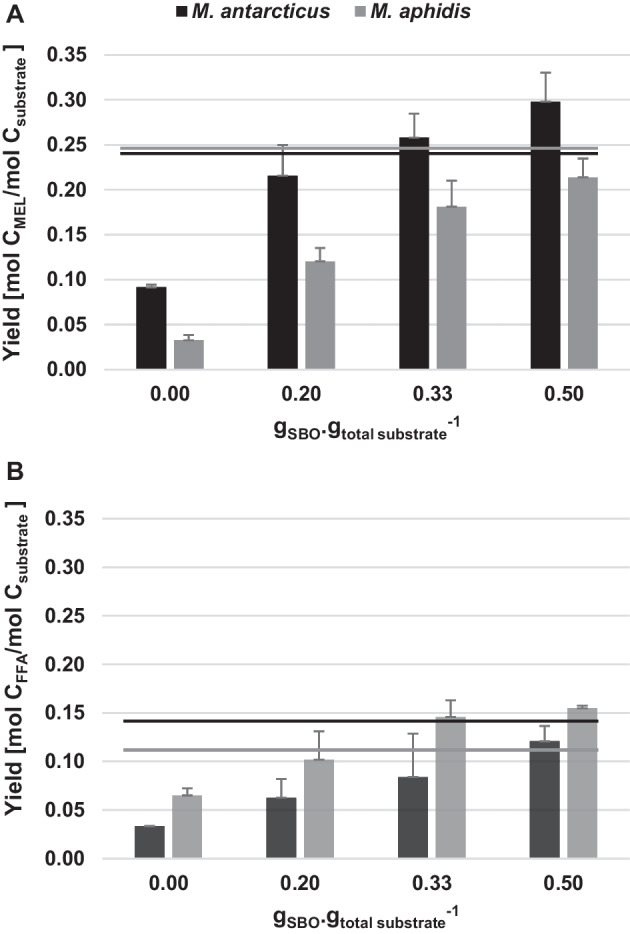
Carbon yields of MEL (**A**) and residual lipids (**B**) in *M. antarcticus* and *M. aphidis* after 14 days cultivations using a total 4 M of carbon in substrate: D-glucose and feeding at day 4 with D-glucose and/or SBO. Lines represent yield obtained in *M. antarcticus* (black line) and *M. aphidis* (grey line) when cultivated with SBO as sole carbon source (4 M in carbon)

The use of D-glucose resulted in relatively low MEL titres if compared to SBO, in equimolar amounts of carbon. The low level of residual lipids observed may be related with cellular synthesis and not from external addition. In *M. antarcticus* cultures, the increase of D-glucose from 80 to 120 g/L slightly increased MEL titres: from 5 to 7 g/L (Table 2). In *M. aphidis* cultures, no differences on MEL titres were observed in such D-glucose concentrations (Table 2) (Fig. 3).

**Table 2:** *Moesziomyces antarcticus* and *M. aphidis* cultivation using co-substrate strategy (bold) as alternative to D-glucose (Glu) or soybean oil (SBO) as sole carbon source, at 2.6 and 3.9 M of total carbon: yields, titres, maximum dry biomass, and productivity

Condition (SBO feeding, g/L)	C (*M*_Glu + SBO_)	MEL (g/L)	Residual lipids (g/L)	Purity (g/g)
***M. antarcticus***				
0	2.6	5.1 ± 0.6	1.9 ± 0.2	0.73
**20**		14.4 ± 0.6	0.9 ± 0.3	0.94
40		14.0 ± 2.8	1.8 ± 0.4	0.89
0	3.9	7.3 ± 0.2	2.1 ± 0.0	0.78
**20**		16.9 ± 2.7	3.9 ± 1.2	0.81
**30**		20.0 ± 2.1	5.1 ± 1.7	0.80
**40**		22.9 ± 2.5	7.3 ± 1.0	0.76
60		18.1 ± 1.7	10.0 ± 0.1	0.64
***M. aphidis***				
0	2.6	3.0 ± 0.2	1.7 ± 0.1	0.64
**20**		11.5 ± 0.2	2.7 ± 0.4	0.81
40		13.2 ± 2.2	2.4 ± 0.6	0.85
0	3.9	2.6 ± 0.5	4.1 ± 0.5	0.39
**20**		12.5 ± 1.5	6.3 ± 1.8	0.66
**30**		14.1 ± 2.3	8.9 ± 0.4	0.61
**40**		16.5 ± 1.6	9.4 ± 0.2	0.64
60		18.7 ± 0.1	8.1 ± 0.2	0.70

Purity (g/g) — ratio of g of MEL to the sum of g of MEL and residual lipids

### Co-substrate Strategy in Mannosylerythritol Lipid Production from *Moesziomyces* spp.

The co-substrate strategy included two set of conditions totalizing 2.3 and 3.9 M of carbon. An initial 1.3 M of carbon, corresponding to 40 g/L of D-glucose, was fed to *M. antarcticus* and *M. aphidis* cultivations, with further supplementation, after 4 days: 1.3 M from 40 g/L of D-glucose or 20 g/L of SBO, or 2.6 M from mixtures of D-glucose and SBO, up to 80 g/L and 40 g/L, respectively.

**Fig. 4:**
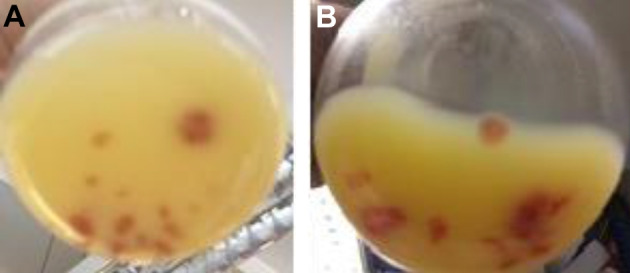
*Moesziomyces aphidis* cultivation using 40 g/L of D-glucose and 20 g/L of SBO after 10 days (**A**) and 14 days (**B**) at 250 rpm and 27 °C

Orange to reddish oil colouring beads were observed at around day 7 (Fig. 4). These beads were MEL-enriched with the presence of residual unconsumed lipids. Interestingly, the beads disappeared over the next days of cultivation of *M. antarcticus*, but not in *M. aphidis* cultivations. The formation of beads might be related with a MEL titre threshold, but also with the interaction of extracellular MEL and residual lipids, since disappearance of beads in *M. antarcticus* did not result in a decrease in the MEL titre in the following days. In this regard, quantification of MEL produced over time was challenging due the heterogeneity of fermentation broth. Therefore, the values of MEL (14 day) were obtained through total extraction of the culture broth with EtOAc (1:1 v/v), twice, and are summarized in Fig. 3 and Table 2.

*Moesziomyces antarcticus* cultivation using D-glucose as sole carbon source, at 2.6 M of carbon, yielded 5.1 g/L of MEL (Table 2). Interestingly, a similar titre of around 14 g/L was obtained when the same 2.6 M of total carbon was fed under the co-substrate strategy, with 20 g/L of SBO, or using SBO as sole source, at 40 g/L. However, the cultivations following co-substrate strategy led to significantly lower values of residual lipids than the ones using SBO as sole carbon source, 0.9 g/L and 1.8 g/L, respectively, increasing the MEL purity up to 94% (Table 2).

MEL titres and yields obtained under co-substrate strategy in *M. aphidis* cultivation with 2.6 M of total carbon were slightly lower if compared with cultivations using SBO as sole carbon source, 11.5 g/L and 13.2 g/L, respectively. Moreover, no improvement on MEL purity was observed using the co-substrate strategy rather than only SBO (Table 2). These results pointed out that there may be no synergistic effects when using substrates with opposite polarities in *M. aphidis* cultivations.

Increasing the total carbon to 3.9 M resulted in the same trend as described before for 2.6 M. Similar or higher MEL titres, but lower residual lipids, were observed in *M. antarcticus* cultivations when the co-substrate strategy was followed (Table 2). A maximum of 22.9 g/L of MEL was obtained for a *M. antarcticus* cultivation using 40 g/L SBO. This corresponds to a yield of 0.30 mol_product_/mol_carbon_ (Fig. 3A) and 0.29 g_MEL_/g_substrate_. This carbon conversion rate decreases to 0.26 in cultivations using 30 g/L of SBO. Both co-substrate conditions, using 30 and 40 g/L of SBO feeding, improved the conversion rate and titre, of 0.24 and 18.1 g/L respectively, observed when SBO was used as sole carbon source, at 60 g/L of SBO. The residual lipids yield decreased from 0.16 mol_lipids_/mol_carbon_, when using SBO as sole carbon source at 60 g/L, to 0.08 and 0.12 when using 30 and 40 g/L of SBO, respectively, in co-cultivation strategy (Fig. 3B).

*Moesziomyces antarcticus* and *M. aphidis* grow and produce MEL from both D-glucose and vegetable oils. Nevertheless, the lipidic fractions of MEL are produced via partial β-oxidation in peroxisomes [31]. In this regard, MEL production from D-glucose should include the activation of the fatty acid biosynthesis pathway after glycolysis, to produce medium to long fatty acyl-chains. The acyl groups will, then, undergo through partial beta-oxidation in the peroxisome [32], to yield the shorter lipidic chains that are incorporated into mannose-erythritol moiety. From the results here obtained, the more efficient MEL production from D-glucose, in *M. antarcticus* when compared with *M. aphidis*, seems to be related with a higher peroxisomal β-oxidation activity. This is supported with the observation of higher lipid accumulation by *M. aphidis*, but lower MEL titres, indicating that although *M. aphidis* can undergo through fatty acid biosynthesis, these medium to long acyl-chains are accumulated in other carbon storage molecules than MEL. Oppositely, the behaviour on MEL production seems to be similar between those strains when vegetable oil was used as sole carbon source.

Generally, the use of different substrates requires different metabolic pathways for intake, degradation, and energy generation. In cell cultivation, the addition of a new substrate might lead to adaptation periods between substrates with consequences on product formation. In this regard, the use of D-glucose followed by the feeding of vegetable oil implies the capacity of the yeasts to incorporate the carbon from this new source. Extracellular lipase activity, previously reported, especially in *M. antarcticus* cultivation on D-glucose [5], is important to shorten the adaptation period on cultivations with feeding of SBO and to the breakdown of the triglycerides fed.

In the rationale behind establishment the co-substrate strategy, D-glucose was used to grow and steady microbial cultures, potentially producing hydrophilic MEL building blocks and/or promoting MEL-genes induction and producing lipases. Nevertheless, further addition of D-glucose is not expected to favour the production of a secondary product such as MEL. Further D-glucose addition increases C/N ratio, known as an important factor for secondary metabolite production, such as reserve lipids or glycolipids. However, MEL production from D-glucose requires de novo MEL-acyl groups building-up, through acetyl Co-A accumulation in the cytosol, and more importantly, fatty acid biosynthesis trough fatty acid synthase complex (FAS), which requires two NADPH molecules per each step of elongation. Then, to be incorporated in mannose-erythritol moiety (ME), these acyl molecules synthesised should undergo partial β-oxidation in the peroxisome. The described metabolism results in a low maximum theoretical MEL production capacity in *Moesziomyces* spp. when using D-glucose. Further supplementation of optimized amounts of vegetable oil, instead of D-glucose, may boost MEL production. The previous production of lipases allows the oil hydrolysis into glycerol and acyl groups, and the latter are incorporated, after partial β-oxidation, in ME to produce MEL, while glycerol and acetyl Co-A contribute for energy balance and mannose and erythritol biosynthesis.

The results obtained illustrate the potential of this promising cultivation strategy attaining high MEL titres while maintaining lower residual lipids, and thus facilitating further downstream processing.

Regarding the downstream processing of MEL, the analysis of the residual lipid content following the co-substrate strategy showed that the residual lipids are mainly free-fatty acids (FFA) and monoglycerols (MG), while triglycerides (TG) and diglycerides (DG) are negligible.

### Improving Mannosylerythritol Lipid Production Sustainable Using Waste Frying Oil as Hydrophobic Carbon source on Co-substrate Strategy

Although SBO has been treated as preferential substrate for MEL production, the use of waste frying oil (WFO) as hydrophobic carbon source to produce MEL in a co-substrate strategy from *M. antarcticus* and *M. aphidis*, may increase process sustainability. The strategy followed the previous discussed protocol with substrate concentrations and additions selected to promote interesting balance of MEL yield and purity. Therefore, after the initial addition of 40 g/L D-glucose, 20 g/L of WFO, instead of SBO, were added at day 4 of cultivation, resulting in addition of a total of 2.7 M of carbon. The parameter analysis of WFO showed that it contains higher peroxide value (58 mEq/kg) than the refined SBO (< 10 mEq/kg), along with a different fatty acid chain composition (WFO major fatty acid is oleic acid, 18:1, while SBO is linoleic acid 18:2) (Table S1, supplementary data).

**Fig. 5:**
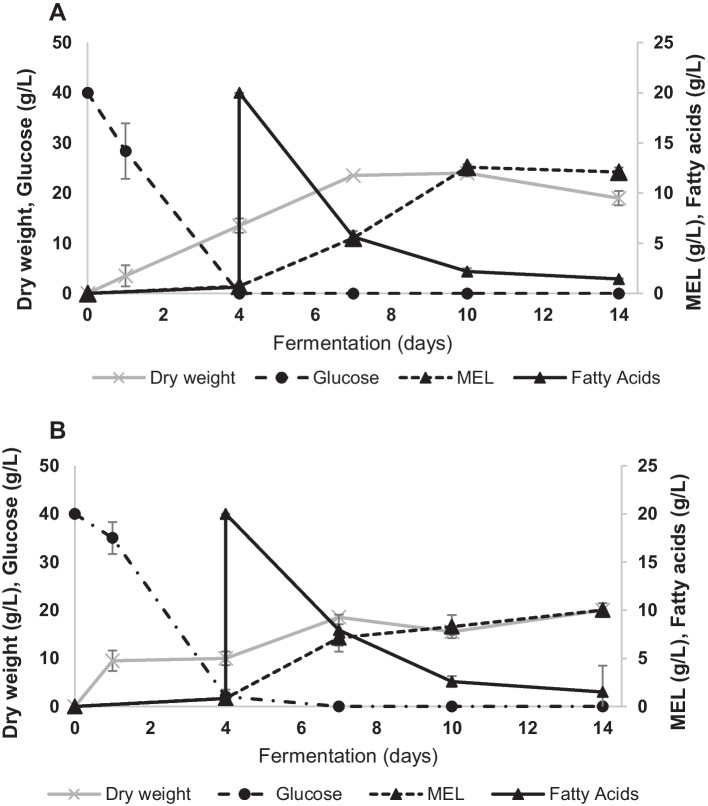
Cultivation of *M. antarcticus* (**A**) and *M. aphidis* (**B**) using 40 g/L D-glucose supplemented with 20 g/L WFO after 4 days of cultivation. Dry biomass (square), D-glucose (circle), MEL (triangle with dashed line), and residual lipids (triangle with full line)

MEL maximum titres of 12.6 and 10.0 g/L were observed at days 10 and 14, for *M. antarcticus* and *M. aphidis*, respectively (Fig. 5). Residual lipids determined were relatively low after 14 days, around 1.5 g/L and 3.4 g/L in *M. antarcticus* and *M. aphidis*, respectively.

The utilization of WFO in co-substrate strategy resulted in MEL titres of around 15% lower than the ones obtained when using SBO under the same co-substrate conditions (Table 3). The use of WFO as sole carbon source (total 4.5 M of carbon) rendered a decrease of around 40% of MEL production when compared to same cultivation conditions using SBO (data not shown). In this regard, the co-substrate strategy may have a positive impact on *Moesziomyces* spp. cultivation due to (i) the first growth phase be ensured by the hydrophilic carbon source (e.g. D-glucose) instead of an more inhibitory carbon source (such as WFO with an high peroxide level of 58 mEq/kg); (ii) in the second stage, when WFO is added to the cultivation well established, the production of the secondary metabolite MELs is only slightly affected (if compared with vegetable oils). Such findings open perspectives on broadening the use of different residual oils in bioprocesses for MEL production.

**Table 3:** Kinetic fermentation parameters of cultivation of *M. antarcticus* and *M. aphidis* using 4% (w/v) D-glucose supplemented with 2.1% (w/v) WFO after 4 days of cultivation

	Condition (WFO feeding, g/L)	C (*M*_Glu + WFO_)	MEL (g/L)	Residual lipids (g/L)	Purity (g/g)
*M. antarcticus*					
WFO	20 g/L	2.6	12.1 ± 0.5	1.5 ± 0.1	0.89
SBO			14.4 ± 0.6	0.9 ± 0.3	0.94
*M. aphidis*					
WFO	20 g/L	2.6	10.0 ± 0.1	3.4 ± 2.7	0.75
SBO			11.5 ± 0.2	2.7 ± 0.4	0.81

Purity (g/g) — ratio of g of MEL to the sum of g of MEL and residual lipids

### Mannosylerythritol Lipid Recovery Through Diafiltration Technology

The previous sections are intrinsically related with costs reduction strategies in MEL production: development of more efficient bioprocesses; possible use of cheap and waste and renewable substrates. Also, accounting for MEL production costs, the downstream processing, usually requires multiple steps and its cost and complexity are highly related with the application of the product. For some industrial applications, a high purity grade is not necessary, and thus, purification costs are reduced. In this sense, the approach of co-substrate can be, by itself, an interesting strategy to achieve relatively high product purities (around 85–90%) and so, for some industrial applications, a high purity grade is not necessary, and thus, purification costs may be reduced. For other, where higher levels of purity are needed, the development of efficient downstream processes is of paramount importance.

The differential of molecular weights of FFA often found in vegetable oils, or FFA and MG driven from triglycerides hydrolysis and MEL was explored for further MEL separation and purification. Therefore, a home-made organic solvent nanofiltration (OSN) membrane made of PBI was prepared. Previous studies reported the PBI preparation from DMAc solutions and crosslinked using α,α′-dibromo-p-xylene (DBX). The use of a 26-wt% PBI solution resulted on previously reported rejections higher than 95% for chlorhexidine (505 Da) in filtrations of EtOAc, but also relatively high at 50–65% for 4-chloroaniline (127 Da) were reported [33]. Rejections for PEG2000 in acetonitrile are reported to be higher than 95% in some studies for 17 wt% PBI membrane. However, other study [34] requires dopes with higher PBI concentrations (22 wt%) to reach levels of rejection for the same solute/solvent system. Therefore, on this preliminary study, a membrane casted from a 22-wt% PBI solution on DMAc was used, to retain MEL, and allow permeation of FFA. Importantly, the prepared PBI membrane was not cross-linked. The membrane permeability was initially characterized through filtrations at 20 bar of acetonitrile solutions, at 24 L/h/bar^1^/m^2^), estimating the membrane rejections for polystyrene of 580 Da and Rose Bengal, a dye of 1017 Da, in acetonitrile at values of 91% and 100%, respectively. This result confirms the membrane selectivity as promising for the application envisaged.

A mixture of MEL and FFA driven from the fermentation, when dissolved in EtOAc, was then used for estimation of membrane rejection at 15 bar at values of 89 ± 1.8% for MEL and 57.9 ± 5.1% for FFA and fresh EtOAc solvent flux of 233.2 ± 5.7 L/m^2^/h. These values point out that the use of nanofiltration on diafiltration mode, to retain MEL while permeating the FFA, will be challenging [35]. For illustrative purposes, a case study of relatively higher difficulty was selected, the EtOAc diafiltrations with the OSN membrane were fed with a 66% purity MEL crude, corresponding to the central value of *M. antarcticus* cultures using SBO alone as carbon source at concentration from 20 to 80 g/L. The diafiltration process was operated until 6 diavolumes, with samples taken at the end and at 3 diavolumes.

The composition of the collected diafiltrate after 3 diavolumes revealed a purity of MEL increased from 66 to 93%, with around 76% of the initial MEL kept in the retentate. Moreover, after 6 diavolumes, the MEL purity increased to 96%, but with remaining 69% of initial MEL in the retentate (Table 4). The assay here performed aims to illustrate the potential of the use of diafiltration, but values obtained are definitely still under optimized with a wide of opportunities to improve membrane process performance. The MEL can be attributed to the membrane rejection and diavolumes used. To increase MEL rejection, developments on membrane manufacturing are required, namely increasing the PBI concentration in the dope or cross-linking the membrane. Alternative strategies to reduce MEL losses include the use of membrane cascades [34]. However, the lower the concentration of residual lipids present on the initial solution, the lower number of diavolumes needed to achieve high MEL purity. Therefore, the diafiltration strategy here suggest for MEL purifications after a simple EtOAc extraction step would be less challenging for culture conditions that yield lower final residual lipids content, preferable FFA, as the MG have higher molecular size, such as the ones obtained with co-substrate strategy and *M. antarcticus* at a 2.7-M total carbon and a 0.35-g_SBO_.g_total substrate_^1^ (see previous section, Table 2). For such case study, considering the under optimized membrane, respective rejections for MEL and FFA, one could calculate [36] a diafiltration using a diavolume of 2 to yield an analytical grade MEL (97% purity) with MEL losses lower than 20%.

**Table 4:** Analysis of MEL and residual lipids in ethyl acetate extracts from diafiltration with PBI membrane

Parameter	MEL (g/L_EtOAc_)	Residual lipids (g/L_EtOAc_)	Purity (g/g)
Feed	1.60 ± 0.00	0.84 ± 0.00	0.66
3DV final retentate	1.21 ± 0.02	0.09 ± 0.00	0.93
6DV final retentate	1.11 ± 0.00	0.05 ± 0.05	0.96

Purity (g/g) — ratio of g of MEL to the sum of g of MEL and residual lipids

Overall, diafiltration is here presented as an alternative industrial downstream processing strategy to extractions procedure, which can reach higher MEL purities [18, 21], but at expenses of using multiple steps using different solvents mixtures which are challenging to recovery. The use of an OSN membrane on diafiltration mode will be particular adequate to purification of MEL contaminated with FFA, and it allows to process the MEL on the same solvent used for culture broth extraction, EtOAc (Bp 77.1 °C, Vp 73 mmHg at 20 °C, ΔH°vap 35 kJ/mol) facilitating further solvent recycling [37].

## Conclusion

Over the years, MEL have been gained attention of industry due to their promising biochemical properties. However, currently, there is no bioprocess with sufficiently high MEL production yields and downstream process efficiencies to reach attractive costs. Therefore, a co-substrate strategy, using a hydrophilic carbon source for initial cellular growth and feeding a hydrophobic carbon source in a second step, was used to foster MEL production by *M. antarcticus* and *M. aphidis*. For *M. antarcticus*, such strategy allowed to reach production of similar MEL titres to the ones obtained using vegetable oil as sole carbon source, but maintaining a low concentration of residual lipids, thus facilitating further downstream processing. Furthermore, a new downstream route was developed to separate and purify MELs from residual lipids by using a homemade flat-sheet organic solvent membrane. The MEL purity can be significantly improved combining the application of diafiltration and cultivation conditions leading to final low levels of residual lipids. The development of membranes with higher retention towards MEL, and/or lower retention towards residual lipids, is desirable to optimize separation and mitigate MEL losses. Future work can include multi-objective optimization techniques to improve biosurfactant production and downstream processing, and thus to further reduce costs of the overall MEL production enabling commercial applications.

### Supplementary Information

Below is the link to the electronic supplementary material.

Supplementary file1 (DOCX 29 KB)

## Data Availability

Data is available on request from the authors.
