# Supplementary material for: Substrates of Opposite Polarities and Downstream Processing for Efficient Production of the Biosurfactant Mannosylerythritol Lipids from Moesziomyces spp
Source: Appl Biochem Biotechnol. 2023 Feb 22;195(10):6132–49. doi: 10.1007/s12010-023-04317-z (PMC10511570; doi:10.1007/s12010-023-04317-z)
Supplement: Supplementary file 1 — Supplementary file1 (DOCX 29 KB) [file 12010_2023_4317_MOESM1_ESM.docx]

# Table S1 – Composition and characterization of the WFO

| **Parameter** | **WFO** |
| --- | --- |
| Acid value (mg KOH/g) | 4.67 |
| Moisture and volatile matter content (% m/m) | 0.10 |
| Insoluble impurity content (% m/m) | < 0.01 |
| Saponification value (mg KOH/g) | 196 |
| Iodine value (g I_2_/100g) | 106 |
| Unsaponifiable matter (% m/m) | n.a. |
| Peroxide value (meq O_2_/Kg) | 58.2 |
| **Fatty acid chain** | **----** |
| C14:0 | 0.1 |
| C16:0 | 4.9 |
| C16:1 | 0.1 |
| C18:0 | 0.1 |
| C18:1 | 62.8 |
| C18:2 | 27.9 |
| C18:3 | 1.5 |
| C20:0 | 0.4 |
| C22:0 | 0.8 |
| C24:0 | n.d. |
| Others | 1.4 |
| Saturated | 6.3 |
| Unsaturated | 92.3 |
